# Medical Textile Stain Detection Based on Chemically Enhanced Visualization and Deep Semantic Segmentation

**DOI:** 10.3390/jimaging12080380

**Published:** 2026-08-13

**Authors:** Wenjie Min, Junfeng He, Zhenping Wan, Jinde Chen, Zhixiang Zou, Yuandong Mo

**Affiliations:** 1 School of Mechanical and Automotive Engineering, South China University of Technology, Guangzhou 510641, China; wenjiemin3@gmail.com (W.M.); zhpwan@scut.edu.cn (Z.W.); 2 School of Intelligent Manufacturing Engineering, Guangdong Polytechnic Normal University, Guangzhou 510665, China; 3 Guangdong Zhensheng Technology Group Co., Ltd., Meizhou 514799, China; 4 School of Electromechanical Engineering, Lingnan Normal University, Zhanjiang 524048, China

**Keywords:** medical textile, chemically enhanced visualization, semantic segmentation, stain detection, visual sorting

## Abstract

Pre-wash sorting of medical textiles is essential for hospital infection control, yet accurate stain detection remains challenging because visually apparent stains often have blurred boundaries, whereas dried urine stains lack distinguishable optical features. This study proposes a medical textile stain detection method integrating chemically enhanced visualization with deep semantic segmentation. Dimethylaminocinnamaldehyde (DMACA) was used to convert latent urine stains into chemically developed stains with orange–red visual features. Based on the spatial color difference ΔE in the L*a*b* color space, 0.0183 mol/L was selected as the most suitable DMACA concentration among those tested. A dataset of 1974 images was constructed, including blood stains, chemically developed urine stains, medication stains, and uncontaminated textiles. A cascaded preprocessing strategy was applied to enhance stain boundaries and suppress textile texture noise, after which an Enhanced semantic segmentation model incorporating residual feature extraction, multiscale feature fusion, and transfer learning was used for pixel-level recognition. The IoU values for blood stains, chemically developed urine stains, and medication stains were 88.11%, 82.67%, and 89.62%, respectively. The average time required for image preprocessing and network inference was 15.39 ms per image. An input-level ablation comparison showed that DMACA-based color development increased the urine-stain IoU from 3.07% to 86.23%, demonstrating its substantial contribution to latent urine-stain detection. These results support the feasibility of integrating front-end chemical feature enhancement with back-end semantic segmentation for multiclass medical textile stain recognition under the current experimental conditions.

## 1. Introduction

Medical textiles are an essential component of hospital infection-control systems, and their cleanliness is closely related to patient safety and healthcare quality [1]. In standard laundering workflows, pre-wash sorting is a critical step because it determines the subsequent washing strategy and directly affects stain-removal efficiency. Accurate identification and classification of textiles contaminated with blood, body fluids, or medication residues can support the selection of appropriate washing procedures and reduce the risk of cross-contamination caused by improper mixing or handling [2]. However, pre-wash sorting in hospital laundry services is still largely performed manually. This process is labor-intensive, inefficient, and highly dependent on operator experience, fatigue, and visual discrimination ability, making consistent sorting difficult, particularly for small stains and low-contrast contaminated regions [3]. Repeated handling and unfolding of contaminated textiles may also promote the aerosolization of pathogenic microorganisms and increase occupational exposure risks for operators [4]. Therefore, a non-contact, accurate, and automated method for detecting stains on medical textiles is needed.

Medical textile stains originate from different contaminants and exhibit diverse optical and morphological characteristics. Typical examples include blood stains, urine stains, and medication stains. Blood stains and medication stains are generally visible under conventional illumination, but penetration, diffusion, and drying within textile fibers often produce irregular morphologies and blurred boundaries. Their appearance is also strongly influenced by the color and texture of the textile substrate. For example, blood and medication residues on dark green surgical gowns or drapes may appear as low-contrast dark regions that are visually confused with the background texture. Although these stains can be captured using visible-light imaging, accurate segmentation remains difficult because of irregular diffusion boundaries, wrinkles, stitching seams, local shadows, reflections, and textile textures. In contrast, dried urine stains are typically latent and often lack distinct color or texture features under normal illumination. Their pixel distributions may substantially overlap with those of the surrounding textile, making stable edge gradients and discriminative features difficult to obtain. Under such low-signal conditions, detection performance is fundamentally constrained by the information contained in the acquired images, and even advanced deep learning models may fail when the input lacks sufficient target features [5]. Reliable multiclass stain detection must therefore address both the blurred boundaries of visually apparent stains and the insufficient visual information of latent urine stains.

Machine vision has been widely investigated for textile surface-defect detection. Early methods mainly relied on handcrafted descriptors, including gray-level co-occurrence matrices, Gabor filters, and edge operators, to extract texture and defect features from textile surfaces. However, their representational and generalization capabilities are often limited under complex textured backgrounds [6,7]. Raheja et al. [8] compared gray-level co-occurrence matrix features with multiscale Gabor filtering for automated fabric-defect detection and showed that the statistical GLCM approach provided favorable accuracy and computational efficiency for conventional texture analysis. Chen et al. [9] developed a Gabor fractal network integrating fractal and grayscale features to detect small fabric defects, including dark yarns, holes, and stains. Boluki et al. [10] proposed a method combining optimized Gabor filtering with adaptive local binarization for real-time defect localization on plain and patterned fabrics. These methods established an important basis for automated textile inspection, but their effectiveness remains dependent on manually designed features and distinguishable target–background differences.

With the development of convolutional neural networks, deep learning has become a major approach to fabric-defect detection. Attention mechanisms, large convolution kernels, multiscale feature extraction, and complementary feature fusion have been introduced to improve feature representation under complex textile backgrounds. Mao et al. [11] incorporated the SimAM attention mechanism into an industrial fabric-defect detection framework to enhance the model’s focus on critical defect features. Zhang et al. [12] combined large convolution kernels, multiscale feature extraction, and a hybrid attention module to strengthen spatial and channel-wise feature representation. Fekri-Ershad and Dehkordi [13] developed a flexible multichannel network integrating texture and spatial information and employed focal loss to improve the learning of difficult samples. Although their method was developed for lung CT analysis, the combination of complementary feature representations and difficult-sample-focused learning provides a methodological reference for jointly modeling textile textures and stain morphologies.

Further advances have been achieved through semantic segmentation, feature-pyramid structures, and multimodal learning. Liu et al. [14] proposed FP-Deeplab, a lightweight encoder–decoder semantic segmentation network incorporating atrous spatial pyramid pooling and a multiscale feature-pyramid module, improving the F1 score and mean intersection over union while reducing model complexity. Jerusha et al. [15] combined a generative adversarial network with CNN-LSTM for multimodal fabric-defect recognition, improving detection accuracy and processing efficiency. Kang et al. [16] developed FD-YOLO by integrating bi-level routing attention and multiscale feature fusion, enabling accurate and real-time detection of multiscale and small fabric defects. Collectively, these studies demonstrate that back-end network optimization can effectively improve detection when the target regions already contain distinguishable color, texture, or morphological features.

However, this prerequisite is not satisfied for many latent stains. Dried urine stains may be optically similar to the textile substrate and lack sufficient contrast, stable edge gradients, and discriminative texture information under visible-light imaging. Under these conditions, improving the back-end network alone cannot fully compensate for the information deficiency at the front-end imaging stage. Enhancing target visibility before image analysis is therefore necessary to provide the segmentation model with sufficiently discriminative input features.

Chemical color development offers a potential solution to this problem. In forensic science and trace-evidence examination, chemical reagents are commonly used to convert latent biological traces into visible colored features [17]. Dimethylaminocinnamaldehyde-based color-development systems can react with urine-related components and generate orange–red products [18,19]. This reaction alters the spectral response of the urine-stained region and transforms a nearly indistinguishable target into a visually detectable feature. From a machine-vision perspective, chemically enhanced visualization can increase target–background contrast and improve the information available for subsequent semantic segmentation. Nevertheless, the integration of front-end chemical feature reconstruction with back-end deep learning for multiclass medical textile stain detection remains insufficiently explored.

To address this gap, this study develops a medical textile stain detection method integrating chemically enhanced visualization with deep semantic segmentation. DMACA-based color development is employed to transform visually indistinct dried urine stains into chemically developed regions with orange–red features, whereas blood stains and medication stains are directly analyzed according to their optical and morphological characteristics. A multiclass dataset containing blood stains, chemically developed urine stains, medication stains, and uncontaminated textiles is constructed across different textile substrates. A cascaded preprocessing strategy is introduced to enhance stain boundaries and suppress textile texture interference, and an Enhanced semantic segmentation model combining residual feature extraction, multiscale feature fusion, and transfer learning is used for pixel-level stain recognition. The study evaluates the feasibility of combining front-end chemical feature enhancement with back-end semantic segmentation under controlled experimental conditions and examines the contribution of DMACA-based color development to latent urine-stain detection. Further workflow-level and field validation is required before the method can be applied to automated pre-wash sorting.

## 2. System Design and Data Acquisition

### 2.1. System Design

**Figure 1:**
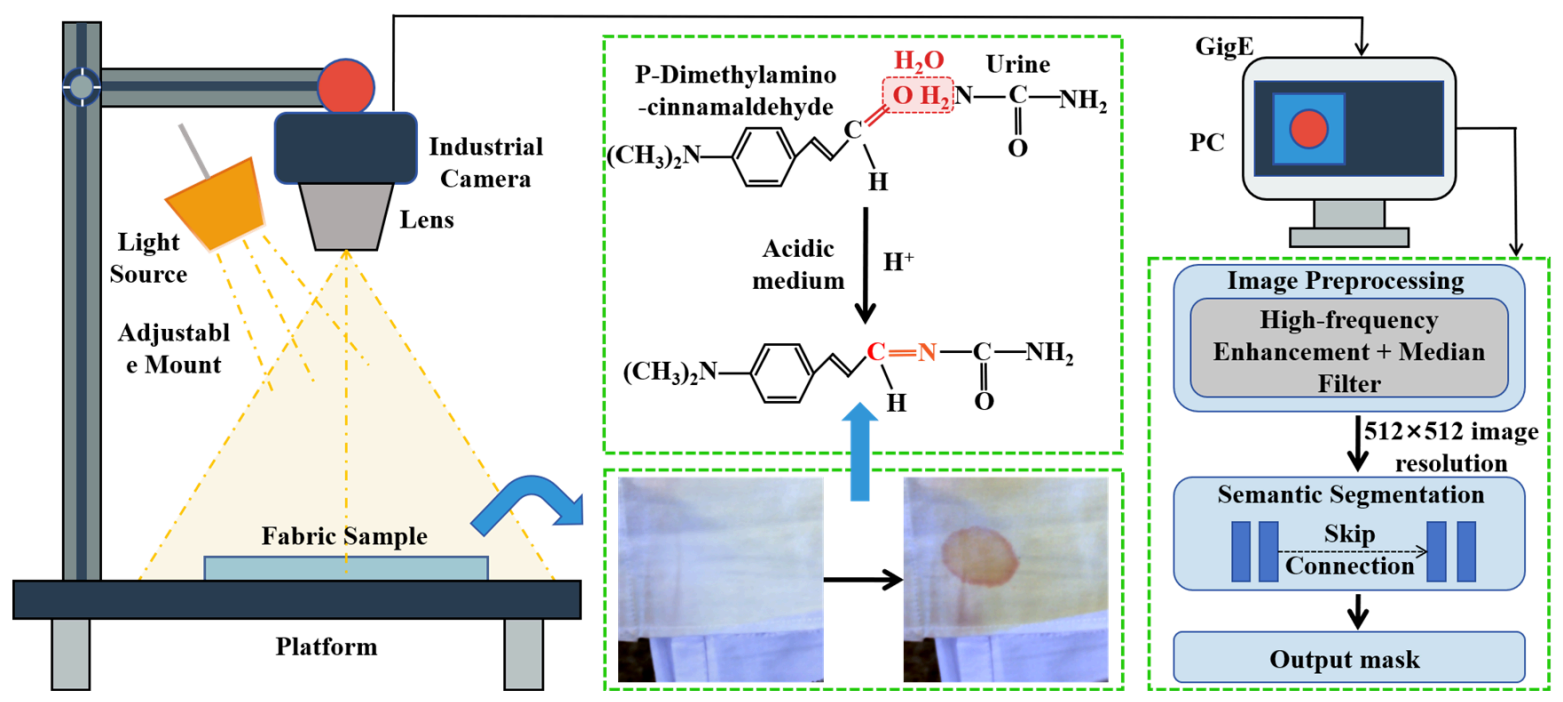
Overall workflow of the proposed framework, including image acquisition, DMACA-based chemical visualization of latent urine stains, cascaded image preprocessing, semantic segmentation, and pixel-level mask output.

The key to medical textile stain detection lies in improving the separability between the target region and the textile background. For latent stains such as urine stains, the primary limitation is the lack of discriminative target features at the front-end imaging stage. For visually apparent stains such as blood stains and medication stains, the main challenges arise from blurred boundaries, irregular morphology, and interference from complex textile textures. To investigate the recognition of multiclass medical textile stains, a detection framework integrating chemical visualization enhancement and deep semantic segmentation was developed in this study. The overall principle and processing workflow are shown in Figure 1.

The system mainly consists of five modules: image acquisition, chemically enhanced visualization, image preprocessing, deep semantic segmentation, and result output. During image acquisition, an industrial vision system was used to obtain surface images of medical textiles. The imaging unit consisted of a 6-megapixel industrial camera (MV-CS060-10GC, Hangzhou Hikrobot Co., Ltd., Hangzhou, China) and an industrial lens with a focal length of 6 mm (MVL-HF0624M-10MP, Hangzhou Hikrobot Co., Ltd., Hangzhou, China). An oblique illumination source was also configured to enhance the visible features of stain regions while reducing the effects of specular reflection and local shadows on image quality. During detection, the medical textile sample was placed on the inspection platform, and stable image acquisition was achieved through trigger control.

For latent urine stains, chemical visualization enhancement was performed before image analysis. After color development, the stained regions exhibited more distinct optical features, thereby improving their separability from the textile background. In contrast, visually apparent blood stains and medication stains were directly processed using the subsequent image-analysis procedure.

The acquired images were then preprocessed to suppress interference from textile textures and enhance the boundary and local-detail features of the stain regions. The processed images were fed into the deep semantic segmentation model. Deep residual feature extraction, multiscale feature fusion, and transfer learning were combined to achieve pixel-level localization and classification of multiple stain categories under complex textile backgrounds. Finally, the segmentation results were used to determine the locations and categories of the stains, providing a methodological basis for automated pre-wash sorting of medical textiles.

### 2.2. Mechanism Analysis of Stain Detection

The proposed system integrates DMACA-based chemical visualization enhancement with deep semantic segmentation to improve stain detectability under complex textile backgrounds. This section presents the system architecture, stain detection mechanisms, and experimental configuration, providing the basis for subsequent preprocessing, dataset construction, and performance evaluation.

#### 2.2.1. Chemical Visualization Enhancement and Feature Reconstruction of Latent Stains

**Figure 2:**
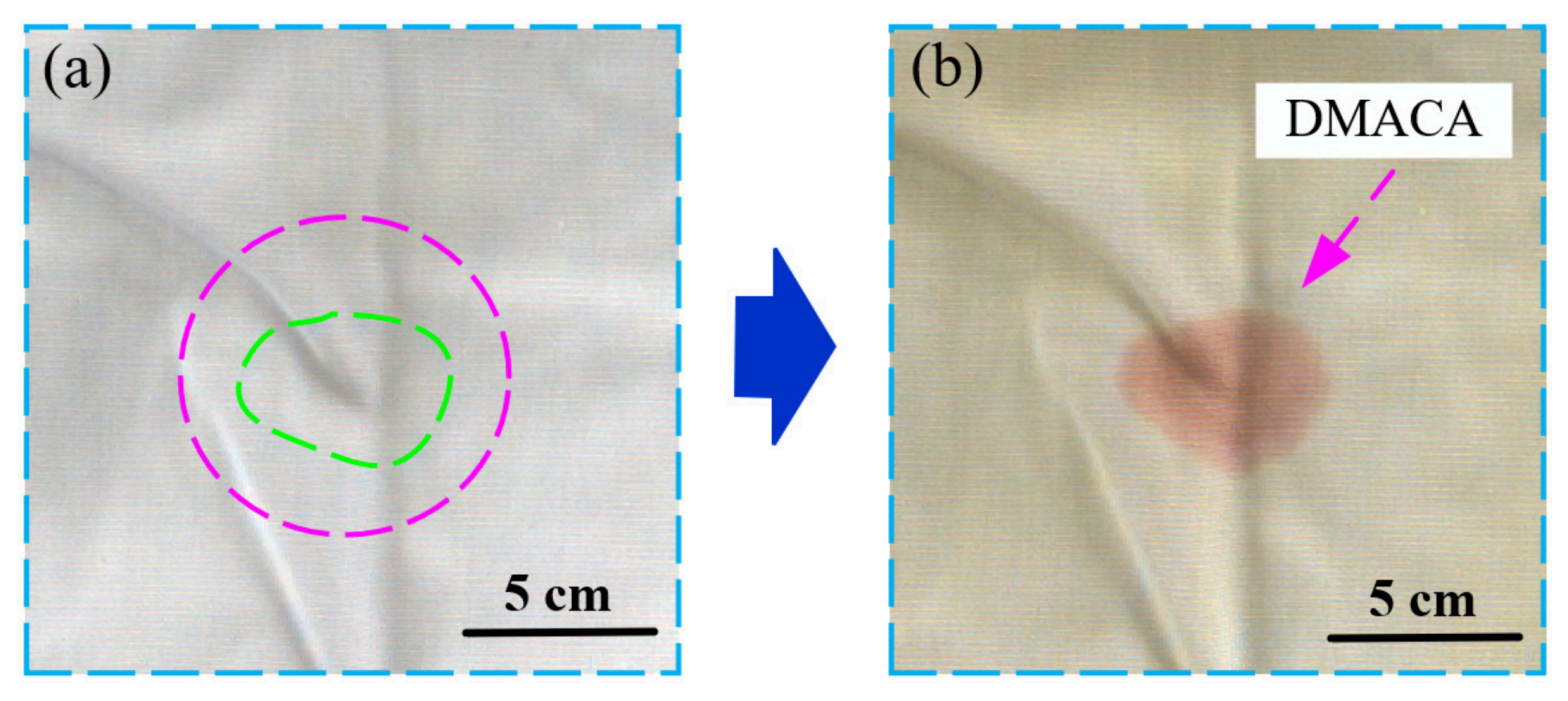
Visual comparison of a latent urine stain before and after DMACA-based chemical color development: (**a**) before color development and (**b**) after color development. The purple dashed circle indicates the approximate location of the urine-stained region revealed after DMACA-based color development, whereas the green dashed contour delineates the actual stain boundary determined from the developed image. Both annotations are shown to indicate the location and extent of the visually indistinct stain before color development.

Dried urine stains usually lack distinct color and texture features under conventional visible-light imaging conditions. Their pixel distribution highly overlaps with that of the textile background, making it difficult to obtain stable edge gradients and discriminative features, as shown in Figure 2. In this study, dimethylaminocinnamaldehyde (DMACA) was used as the color-developing reagent to enhance the optical response of urine-stained regions through a chemical color development reaction. As a result, latent stains with low contrast were transformed into visually detectable features that could be reliably captured by the machine vision system.

Urine contains a certain concentration of metabolites such as urea. In an acidic medium, the aldehyde group in DMACA can be protonated and activated, and then undergo nucleophilic addition and dehydration condensation with the amino group in urea, producing an orange–red protonated condensation product [18]. The reaction process can be expressed as Equation (1).

(1) $${R\text{-}\mathrm{CHO}+H}_{2}{N\text{-}\mathrm{CO}\text{-}\mathrm{NH}}_{2}\overset{H^{+}}{\to }{R\text{-}\mathrm{CH}=N\text{-}\mathrm{CO}\text{-}\mathrm{NH}}_{2}{+H}_{2}O$$

where R represents the dimethylaminocinnamyl group $\left({\left({\mathrm{CH}}_{3}\right)}_{2}{N\text{-}C}_{6}H_{4}\text{-}\mathrm{CH}=\mathrm{CH}\text{-}\right)$.

From the perspective of the color development mechanism, the orange–red product generated by the reaction between DMACA and urea has an extended π-conjugated structure. The dimethylamino group acts as an electron donor, while the protonated imine structure acts as an electron acceptor. Together, they induce a pronounced charge delocalization effect, thereby enhancing the absorption capability of the product in the visible-light region [18,19]. Therefore, DMACA-based color development is not simply a color enhancement process; rather, it actively reconstructs the target features of latent stains before image acquisition. This process changes the spectral response characteristics of the urine-stained region and converts low-contrast contaminated areas that are originally difficult to distinguish into visible colored regions with clear optical differences.

After color development, the color difference between the urine-stained region and the textile background increased. The target boundary and spatial distribution became clearer, providing more discriminative input features for the subsequent semantic segmentation model.

To determine a stable DMACA concentration suitable for machine-vision detection, six concentration levels were evaluated using the spatial color difference ΔE between the chemically developed urine-stain region and the reagent-only background region in the L*a*b* color space. Detailed information regarding reagent preparation, region-of-interest selection, L*a*b* feature extraction, and ΔE calculation is provided in Appendix A.

**Figure 3:**
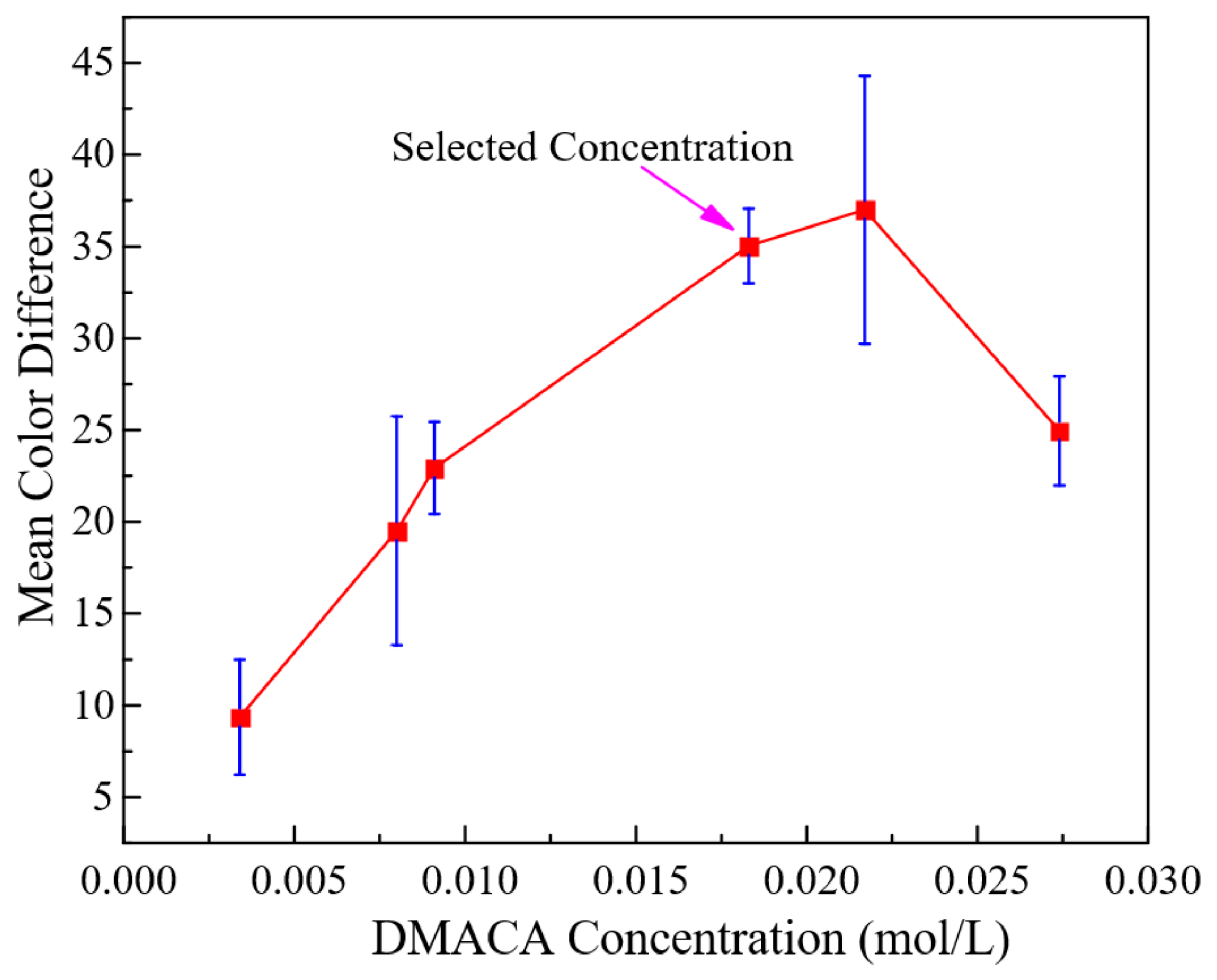
Color-difference response curves of urine stains under different DMACA concentrations.

As shown in Table 1 and Figure 3, the spatial color difference ΔE between the urine color-developed region and the background region first increased and then decreased with increasing DMACA concentration. At low concentrations, the color development reaction was insufficient, resulting in limited color separation between the urine-stained region and the background. At excessively high concentrations, yellowing of the reagent background and enhanced local diffusion increased the background response and sample-to-sample variability. Although group E (0.0217 mol/L) showed the highest mean color difference of 37.000, its standard deviation reached 7.301, indicating insufficient response stability. In comparison, group D (0.0183 mol/L) maintained a relatively high color difference of 35.035 while exhibiting the lowest standard deviation of 2.040, demonstrating better color separation capability and response consistency.

Considering color contrast, response stability, and compatibility with the textile substrate, group D at 0.0183 mol/L was selected as the most suitable concentration among those tested. This concentration consistently transformed latent urine stains that were difficult to identify under conventional imaging into orange–red visual features with pronounced contrast. Thus, front-end feature reconstruction of latent stains was achieved, providing a reliable visual input for the subsequent deep semantic segmentation model.

#### 2.2.2. Feature Representation and Semantic Segmentation of Visually Apparent Stains

Unlike latent urine stains, whose detection is primarily limited by insufficient visual information at the front-end imaging stage, visually apparent stains such as blood stains and medication stains can be directly captured using visible-light imaging. However, their accurate recognition remains affected by fabric color, fiber texture, stain diffusion morphology, and blurred boundaries.

From an optical perspective, the separability of visually apparent stains varies considerably across textile substrates of different colors. On light-colored substrates, such as white medical work uniforms and blue nursing uniforms, the red features of blood stains and the yellow–brown features of iodophor stains generally provide relatively high contrast against the background, thereby offering clear color-gradient information for feature extraction. On dark substrates such as green surgical gowns, however, the optical contrast between the stains and the background is reduced. Blood stains and medication stains may therefore appear as similar low-contrast dark regions, decreasing interclass differences and increasing the risk of confusion during pixel-level classification.

From a morphological perspective, blood stains and medication stains are liquid contaminants that form irregular boundaries as they penetrate, diffuse, and dry within the warp and weft fibers of the textile. Medication stains may exhibit a nonuniform distribution characterized by darker edges and a lighter center, resembling the coffee-ring effect caused by solute migration toward the stain boundary during evaporation. These characteristics, including nonuniform color distributions, discontinuous boundaries, and substantial variations in stain size and shape, make reliable segmentation difficult using conventional methods based on color thresholds, edge gradients, or simple contour extraction. In addition, warp and weft textures, stitching seams, wrinkle-induced shadows, and local reflections further interfere with boundary extraction and increase the difficulty of pixel-level recognition.

Automated pre-wash sorting of medical textiles requires both the localization of contaminated regions and the identification of stain categories. Therefore, multiclass stain detection was formulated as a pixel-level semantic segmentation task. Specifically, each image pixel was assigned to one of four classes: background, blood stain, chemically developed urine stain, or medication stain. Unlike image-classification methods that provide only image-level labels, semantic segmentation simultaneously provides the location, shape, and boundary of each contaminated region, making it more suitable for stains with blurred boundaries and irregular morphologies.

**Figure 4:**
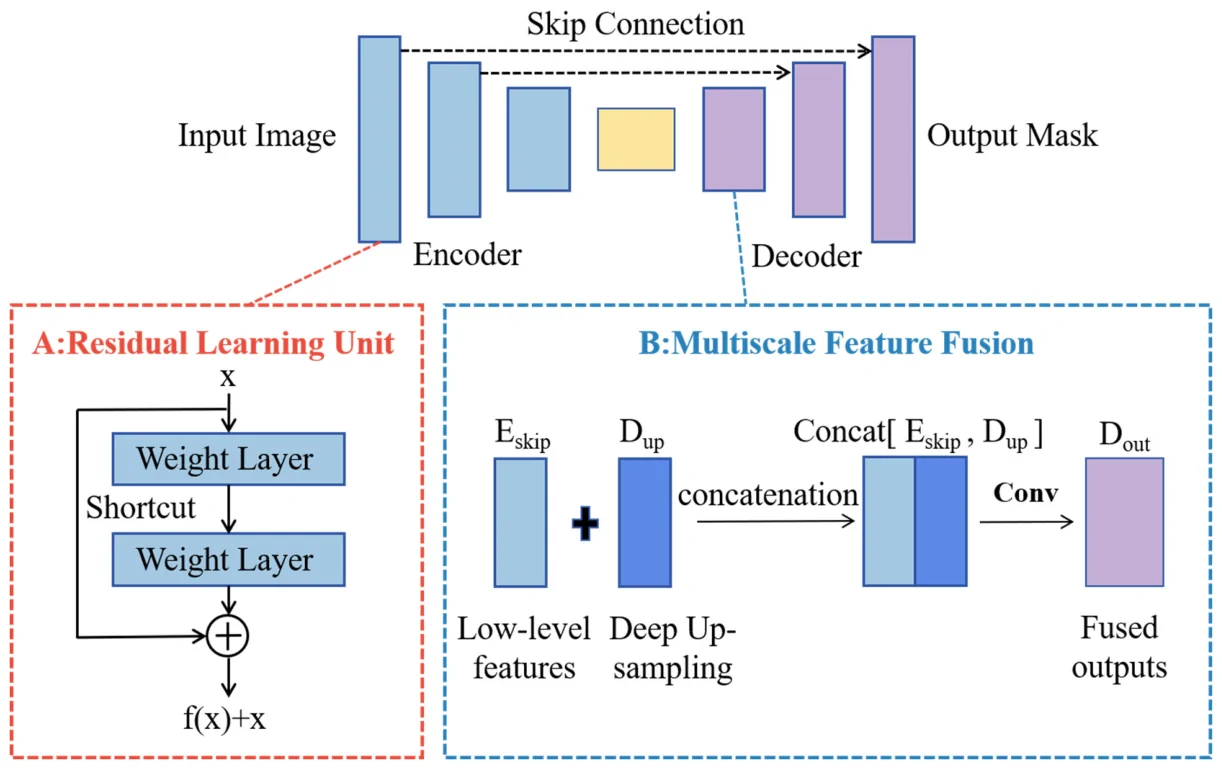
Conceptual illustration of pixel-level semantic segmentation for medical textile stain recognition, including the overall encoder–decoder workflow, (**A**) the residual learning unit, and (**B**) the multiscale feature fusion unit. The diagram illustrates the general segmentation principle rather than the complete proprietary architecture of the HALCON Enhanced model. The yellow square denotes the bottleneck feature representation between the encoder and decoder, containing the high-level semantic features passed to the decoder for spatial-resolution recovery.

To explain the general mechanism of pixel-level prediction, semantic segmentation was first represented using a conceptual encoder–decoder fully convolutional network framework [20,21]. The encoder extracts hierarchical semantic features through convolution and downsampling, whereas the decoder progressively restores spatial resolution through upsampling and feature fusion to generate pixel-level segmentation masks, as illustrated in Figure 4. This schematic explains the general segmentation principle and does not reproduce the complete proprietary architecture of the HALCON Enhanced model.

The residual learning unit (A) and multiscale feature fusion unit (B) in Figure 4 serve only to illustrate the mechanisms through which deep segmentation networks can improve feature representation and boundary recovery under complex textile backgrounds. In the conceptual encoder, residual learning uses shortcut connections to perform identity mapping, thereby alleviating vanishing gradients and network degradation during deep-network training. This structure supports more stable learning of stain boundaries, local color variations, texture differences, and low-contrast regions [22]. The residual learning process is expressed in Equations (2) and (3).

(2) F(x)=H(x)−x

(3) $$y=\sigma (F(x,\{W_{i}\})+x)$$

where x is the input of the residual module, H(x) is the desired underlying mapping, F(x) is the fitted residual mapping, σ is the nonlinear activation function, $F(x,\{W_{i}\})$ is the residual function to be learned in the i-the residual block, and $W_{i}$ denotes the set of convolutional-layer weights in the i-the residual block.

Although repeated downsampling strengthens high-level semantic representation, it also reduces spatial resolution and may result in the loss of fine boundary information. To improve boundary recovery, the conceptual decoder employs multiscale feature fusion. Shallow encoder features containing spatial details are fused with upsampled deep decoder features through skip connections. This process combines category-level semantic information with local boundary details, improving the representation of irregular stain edges, low-texture regions, and complex background interference. The feature-fusion process is expressed in Equation (4).

(4) $$D_{out}=\;\mathrm{Conv}\left(\left[D_{up},E_{skip}\right]\right)$$

where $D_{out}$ is the fused output feature, $D_{up}$ is the upsampled feature from the previous decoder layer, $E_{skip}$ is the same-level feature from the encoder, $\left[D_{up},E_{skip}\right]$ denotes the channel-wise concatenation of $D_{up}$ and $E_{skip}$, and Conv denotes the convolution operation used for feature fusion.

During training, a multiclass cross-entropy loss function was used to optimize the model parameters based on the pixel-level predictions [23]. For each pixel, the network generated a probability distribution over four classes: background, blood stain, chemically developed urine stain, and medication stain. The difference between the predicted distribution and the manually annotated ground-truth label was then quantified using cross-entropy loss, as expressed in Equation (5).

(5) $$L=-\frac{1}{N^{\prime }}\sum_{i=1}^{N}\sum_{c^{\prime }=1}^{C}y_{i,c^{\prime }}\log(p_{i,c^{\prime }})$$

where $N^{\prime }$ is the total number of pixels in the input image, C is the total number of segmentation classes, $y_{i,c^{\prime }}$ is the ground-truth label distribution of pixel i for class $c^{\prime }$, represented by one-hot encoding, and $p_{i,c^{\prime }}$ is the predicted probability output by the network after Softmax activation.

The above framework provides the conceptual basis for pixel-level stain segmentation. The available architectural information for the HALCON Enhanced model used in this study is described below.

According to the relevant literature [24], the Enhanced semantic segmentation model available in MVTec HALCON uses ResNet-50 as the feature-extraction backbone and incorporates a pyramid pooling module and a Fire module. The input image is processed through convolutional, pooling, and residual structures to extract hierarchical semantic features. The resulting feature maps are then passed through a pyramid pooling module with pooling scales of 6 × 6, 3 × 3, 2 × 2, and 1 × 1 to capture contextual information over different receptive fields. The features obtained at each scale are convolved, upsampled, and concatenated to form a multiscale representation that integrates local details with global semantic information.

The concatenated features are further processed by the Fire module for channel compression and feature fusion, followed by convolutional operations that generate a pixel-level segmentation mask with the same spatial dimensions as the input image. In this study, the pretrained Enhanced semantic segmentation model available in MVTec Deep Learning Tool 24.05 was used for initialization and subsequently fine-tuned on the medical textile stain dataset. Because its internal implementation is encapsulated within commercial software, the architectural description presented here is based on the relevant literature, information available from MVTec, and configuration details that could be confirmed from the actual training project.

### 2.3. Experimental Setup

The experiments were conducted using the multiclass medical textile stain image dataset constructed in this study, which contained 1974 images. The dataset comprised 569 blood-stain images, 592 medication-stain images, 599 chemically developed urine-stain images, and 214 uncontaminated-textile images. The distribution of each image category across the three textile substrates is presented in Table 2. 

The dataset was divided into training, validation, and test sets in a ratio of 70:15:15. The pretrained Enhanced semantic segmentation model was used for initialization to improve convergence stability and facilitate feature transfer under limited-data conditions. The Adam optimizer was used, and the initial learning rate was set to 1 × 10^−4^. Stepwise learning rate decay was performed at the 50th and 75th epochs to reduce the parameter update magnitude in the later training stage and promote stable model convergence. To address the class imbalance caused by the number of background pixels being significantly larger than that of stain pixels, a class-weight balancing strategy was introduced into the solver.

MVTec Deep Learning Tool 24.05 was used for model training, validation, and evaluation. The training set was used to update the model parameters, whereas the validation set was used to monitor the loss and mIoU during training and assess model convergence. The independent test set was used exclusively for final performance evaluation and was not involved in model optimization.

Data augmentation was applied only to the training set. Augmented samples were generated dynamically according to the predefined settings and were not saved as additional image files. Therefore, augmentation did not alter the original numbers of samples in the training, validation, or test sets. No random augmentation was applied during validation or testing. Except for the convergence analysis performed during training, all reported values of precision, recall, F1 score, IoU, mIoU, and FWIoU were calculated exclusively on the independent test set.

During evaluation, each test image was fed into the trained Enhanced semantic segmentation model to generate pixel-level predictions for four classes: background, blood stain, chemically developed urine stain, and medication stain. The predicted masks were compared pixel by pixel with the corresponding manually annotated ground-truth masks. The numbers of true-positive, false-positive, and false-negative pixels were then calculated for each class and used to determine the semantic segmentation metrics.

Intersection over union (IoU) was used as the primary metric for quantitatively evaluating pixel-level segmentation performance. Precision, recall, F1 score, and frequency-weighted intersection over union (FWIoU) were also calculated for comprehensive evaluation. For class i, these metrics are defined in Equations (6)–(9).

(6) $$Precision_{i}=\frac{TP_{i}}{TP_{i}+FP_{i}}$$

(7) $$Recall_{i}=\frac{TP_{i}}{TP_{i}+FN_{i}}$$

(8) $$F1_{i}=2\times \frac{Precision_{i}\times Recall_{i}}{Precision_{i}+Recall_{i}}$$

(9) $$IoU_{i}=\frac{TP_{i}}{TP_{i}+FP_{i}+FN_{i}}$$

where TP is the number of pixels correctly predicted as class i, $FP_{i}$ is the number of pixels from the background or other classes incorrectly predicted as class i, and $FN_{i}$ is the number of pixels belonging to class i that are not correctly predicted by the model.

To evaluate the overall multiclass segmentation performance, the mean F1 score (Mean_F1) and mean intersection over union (mIoU) were calculated using Equations (10) and (11).

(10) $$\mathrm{Mean}\_F1=\frac{1}{C^{\prime \prime }}\sum_{i=1}^{C^{\prime \prime }}F1_{i}$$

(11) $$mIoU=\frac{1}{C^{\prime \prime }}\sum_{i=1}^{C^{\prime \prime }}IoU_{i}$$

Considering the obvious class-area imbalance in medical textile stain detection, FWIoU was further used to evaluate the overall segmentation performance of the model in the complete scene, as defined in Equation (12).

(12) $$FWIoU=\sum_{i=1}^{C^{\prime \prime }}\frac{t_{i}}{\sum_{j=1}^{C^{\prime \prime }}t_{j}}\times IoU_{i}$$

where j is the class index, $C^{\prime \prime }$ is the total number of segmentation classes, $\sum_{j=1}^{C^{\prime \prime }}t_{j}$ is the total number of pixels in the test set, and $t_{i}$ is the number of pixels whose ground-truth label belongs to class i.

The global random seed was fixed at 1, and one complete training run was conducted using the same software and hardware environment and training parameters. Although fixing the random seed helps control stochastic variation during training, it cannot replace multiple independent runs using different random seeds.

Accordingly, the reported mIoU, Mean_F1, FWIoU, and class-specific segmentation metrics were obtained by evaluating the model from this single training run on the independent test set, rather than by averaging the results of multiple runs. Because repeated training using different random seeds was not performed, means, standard deviations, and confidence intervals across multiple runs were not calculated, and no statistical significance analysis was conducted. This represents a limitation of the statistical evaluation in the present study.

The detailed training hyperparameters are listed in Table 3. Data augmentation included random horizontal mirroring, random rotations in 90° increments, global brightness variations of ±20 gray levels, and 20% variations in contrast and saturation. Scaling and noise perturbations were not applied, and all augmentation operations were restricted to the training set.

The average processing time per image was measured on the independent test set to characterize the efficiency of image preprocessing and network inference under the reported hardware configuration. The reported time included image preprocessing and network inference but excluded image acquisition, reagent application, the color-development waiting period, textile handling and transport, and mechanical sorting. The hardware platform consisted of an Intel Xeon Gold 6430 processor, an NVIDIA GeForce RTX 3090 graphics card, and 128 GB of system memory. The software environment was Windows 11, and model development and inference were performed using MVTec Deep Learning Tool 24.05.

## 3. Image Preprocessing and Dataset Construction for Multiclass Stain Detection

To obtain high-quality inputs for training the deep semantic segmentation model, a multiclass medical textile stain image dataset was constructed following image acquisition and chemical color development. A cascaded image preprocessing strategy and an online data augmentation scheme were also implemented. This section comprises three parts. First, the original images were processed to enhance stain boundaries and suppress background noise, thereby improving the local separability of the stain regions. Second, a multiclass dataset with pixel-level annotations was constructed for blood stains, chemically developed urine stains, and medication stains. Finally, the variations in loss and mIoU during a single training run with a fixed random seed were analyzed to characterize model convergence under the reported training configuration.

### 3.1. Cascaded Preprocessing and Target Feature Enhancement Strategy

**Figure 5:**
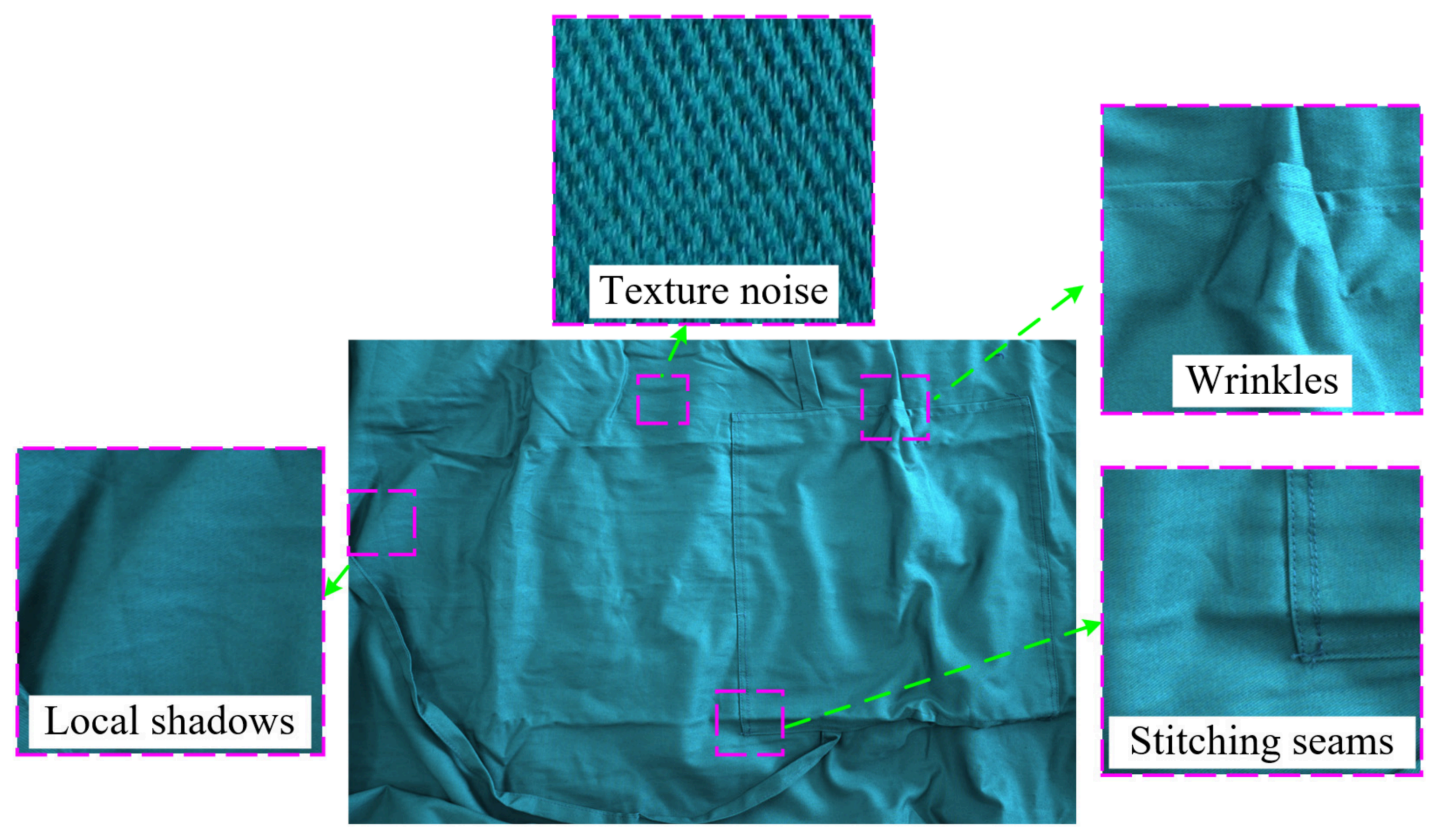
Representative complex background factors in medical textile stain images, including fabric texture, wrinkles, stitching seams, local shadows, and stain regions.

To approximate the practical conditions of medical textile pre-wash sorting, the textile samples were placed on the inspection platform without additional tensioning or flattening during image acquisition. Consequently, background variations such as fabric wrinkles, stitching seams, and local shadows were retained in the original images. To improve acquisition efficiency and maintain consistent illumination and imaging conditions within each batch, a single-field, multi-target acquisition strategy was adopted. Multiple stain regions with different morphologies were prepared on the same textile substrate, and full-field images representing different combinations of textile substrates and stain types were acquired. Representative samples are shown in Figure 5.

Medical textile surfaces contain pronounced warp and weft textures, wrinkle-induced shadows, and local reflections, which may reduce the gradient difference between stain boundaries and the background. To improve the local contrast of the stain regions and generate suitable inputs for semantic segmentation, a cascaded preprocessing strategy consisting of high-frequency enhancement followed by median filtering was employed. High-frequency enhancement was first used to strengthen stain boundaries and local details, whereas median filtering was subsequently applied to suppress amplified textile textures and local image noise. This procedure enhanced the target features while preserving the overall continuity and morphology of the stain regions.

To address the blurred stain-edge problem, a high-frequency enhancement filter was applied to the original images [25]. By extracting the difference component between the original image and its low-frequency smoothed image, high-frequency edge information in the image was enhanced, thereby increasing the local gradient difference between the stain region and the fabric background. The mathematical model is expressed in Equation (13).

(13) $$g(x,y)=f(x,y)+k\left(f(x,y)-f_{low}(x,y)\right)$$

where f(x,y) is the original image, $f_{low}(x,y)$ is the low-frequency image obtained by local mean filtering of the original image, and k is the enhancement coefficient.

In this study, the enhancement coefficient (k) was set to 1.0 through repeated experimental comparisons and parameter optimization using representative stain images. The selection of this value was intended to balance stain-boundary enhancement and background-noise suppression. Specifically, it effectively strengthened the local gradients of low-contrast stain boundaries while avoiding excessive amplification of the fabric warp–weft texture and local imaging noise.

It should be noted that high-frequency enhancement may amplify the fine woven texture and local noise on the textile surface while strengthening stain edges. To reduce the influence of such noise on subsequent feature extraction, median filtering was introduced as a post-processing step [26]. Median filtering is a nonlinear smoothing method that can suppress isolated noise while preserving the boundary structure of stain regions. Its mathematical model is given in Equation (14).

(14) $$h(x,y)=\underset{(s,t)\in S_{xy}}{\mathrm{median}}\{f(s,t)\}$$

where f(x,y) and h(x,y) represent the original image and the output image, respectively, and $S_{xy}$ is the neighborhood window centered at (x,y).

Because stain boundaries generally exhibit irregular diffusion morphologies, a circular filtering mask was used together with mirror-reflection boundary handling. The mask radius was set to 3 pixels to balance noise suppression and boundary preservation. A smaller radius was insufficient to suppress fine textile textures and local noise amplified by high-frequency enhancement, whereas a larger radius caused excessive smoothing and weakened irregular boundaries and local stain details. Comparative experiments showed that a radius of 3 pixels effectively reduced high-frequency noise while preserving the main stain contours; therefore, this value was adopted.

**Figure 6:**
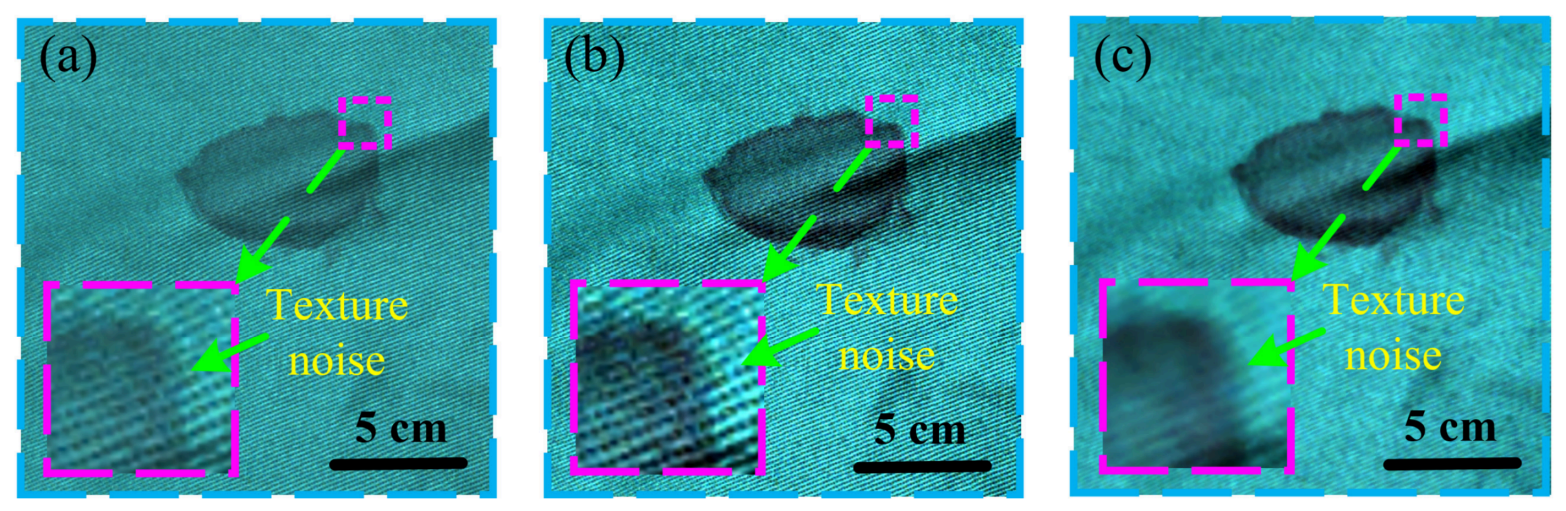
Effect of cascaded preprocessing on fabric background texture and stain features in a medical surgical gown: (**a**) original image, (**b**) high-frequency enhanced image, and (**c**) median-filtered image.

After cascaded preprocessing, regions of interest with a resolution of 512 × 512 pixels were cropped using the crop_part operator according to the manually annotated target center coordinates and used as the input for the subsequent deep semantic segmentation model. Typical preprocessing results are shown in Figure 6. High-frequency enhancement visibly strengthened the boundary features of the stain regions, but also amplified the warp–weft textures on the fabric surface (Figure 6b). After median filtering (Figure 6c), fine-grained texture noise was effectively suppressed, and the main stain contour was largely preserved, providing more stable input features for subsequent model training.

### 3.2. Construction of the Multiclass Stain Dataset and Online Augmentation

To ensure experimental consistency and reproducibility, three types of medical garments commonly used in hospitals were selected as textile substrates: white medical work uniforms, blue nursing uniforms, and green surgical gowns. Animal whole blood, iodophor solution, and urine samples from healthy adults were used to prepare blood stains, medication stains, and urine stains, respectively. Accordingly, multiclass medical textile stain samples covering different textile substrates and stain categories were constructed.

**Figure 7:**
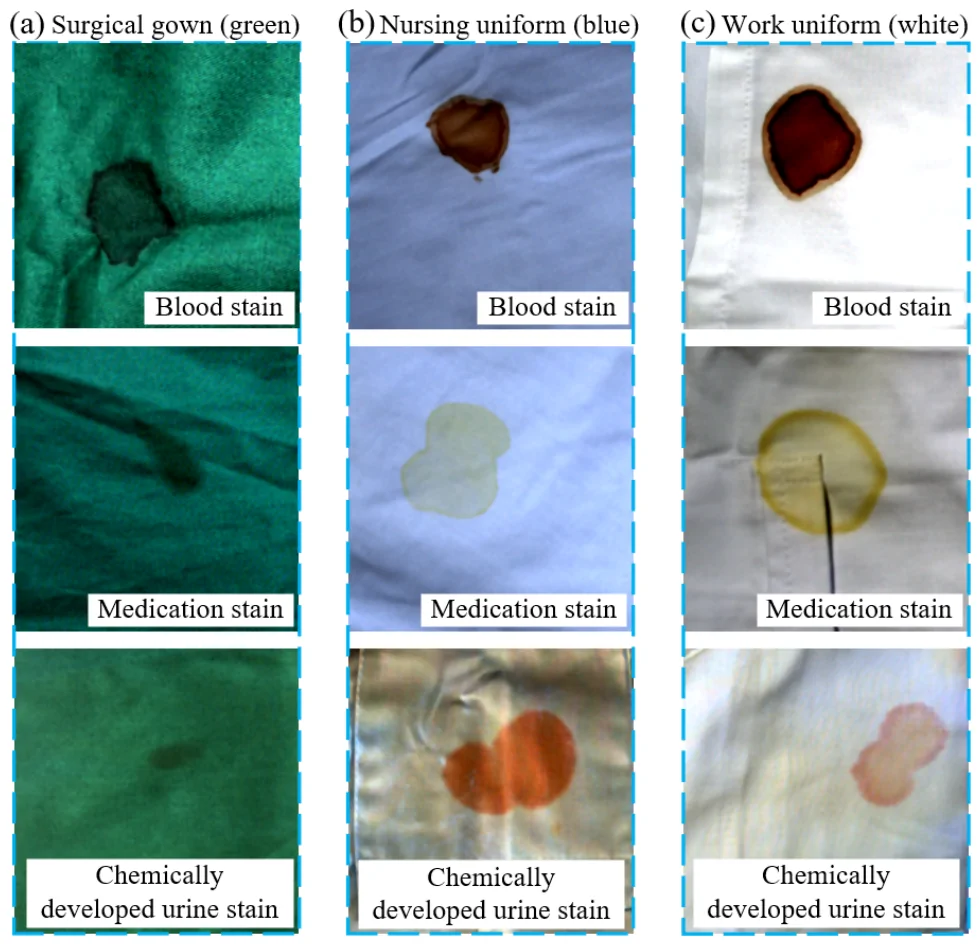
Representative preprocessed samples of the multiclass medical textile stain dataset.

After deposition on the textile surface, all stain samples were allowed to dry naturally for 24 h before subsequent imaging or chemical color development. The dried blood-stain and medication-stain samples were directly used as visually apparent stain data to evaluate model performance under complex backgrounds and blurred-boundary conditions. For the dried urine-stain samples, the DMACA reagent was applied to convert the nearly transparent urine residues into orange–red visual features. These samples were used to evaluate the model’s ability to identify chemically developed urine stains. Representative preprocessed samples from different stain categories are shown in Figure 7.

Manual pixel-level semantic annotation was performed to construct the supervised learning dataset, and a two-stage cross-checking procedure was adopted to ensure annotation quality. First, one researcher carefully delineated the polygonal boundaries of each stain region using an interactive annotation interface and assigned each pixel to one of four classes: background, blood stain, chemically developed urine stain, or medication stain. All pixels in uncontaminated-textile images were labeled as background. A second researcher then independently reviewed all annotations. For samples with highly blurred or ambiguous boundaries, the two annotators jointly revised the boundaries according to the observed stain morphology resulting from diffusion and drying until consensus was reached. The reviewed annotations were converted into single-channel masks with the same dimensions as the original images and used as pixel-level ground-truth labels for model training, validation, and testing.

To reduce the risk of overfitting under the current dataset conditions, on-the-fly data augmentation was applied only to the training set [27]. The augmentation operations included random horizontal flipping, rotations in 90° increments, global brightness variations of ±20 gray levels, and 20% variations in contrast and saturation. Scaling and noise perturbations were not applied. The augmented samples were generated dynamically during image loading and were not saved as additional image files. Therefore, augmentation did not alter the number of samples in the original dataset. No random augmentation was applied to the validation or independent test sets, ensuring that training monitoring and final performance evaluation were based on fixed original samples.

These augmentation operations introduced only limited geometric and photometric variations within the predefined ranges and could not fully represent differences in hospital environments, camera sensors, lens parameters, illumination spectra, textile washing and aging conditions, or stain concentrations. Therefore, training data augmentation cannot substitute for external generalization validation. Model performance under conditions not represented in the current dataset must be evaluated using independently collected external data.

### 3.3. Model Training and Convergence Analysis

**Figure 8:**
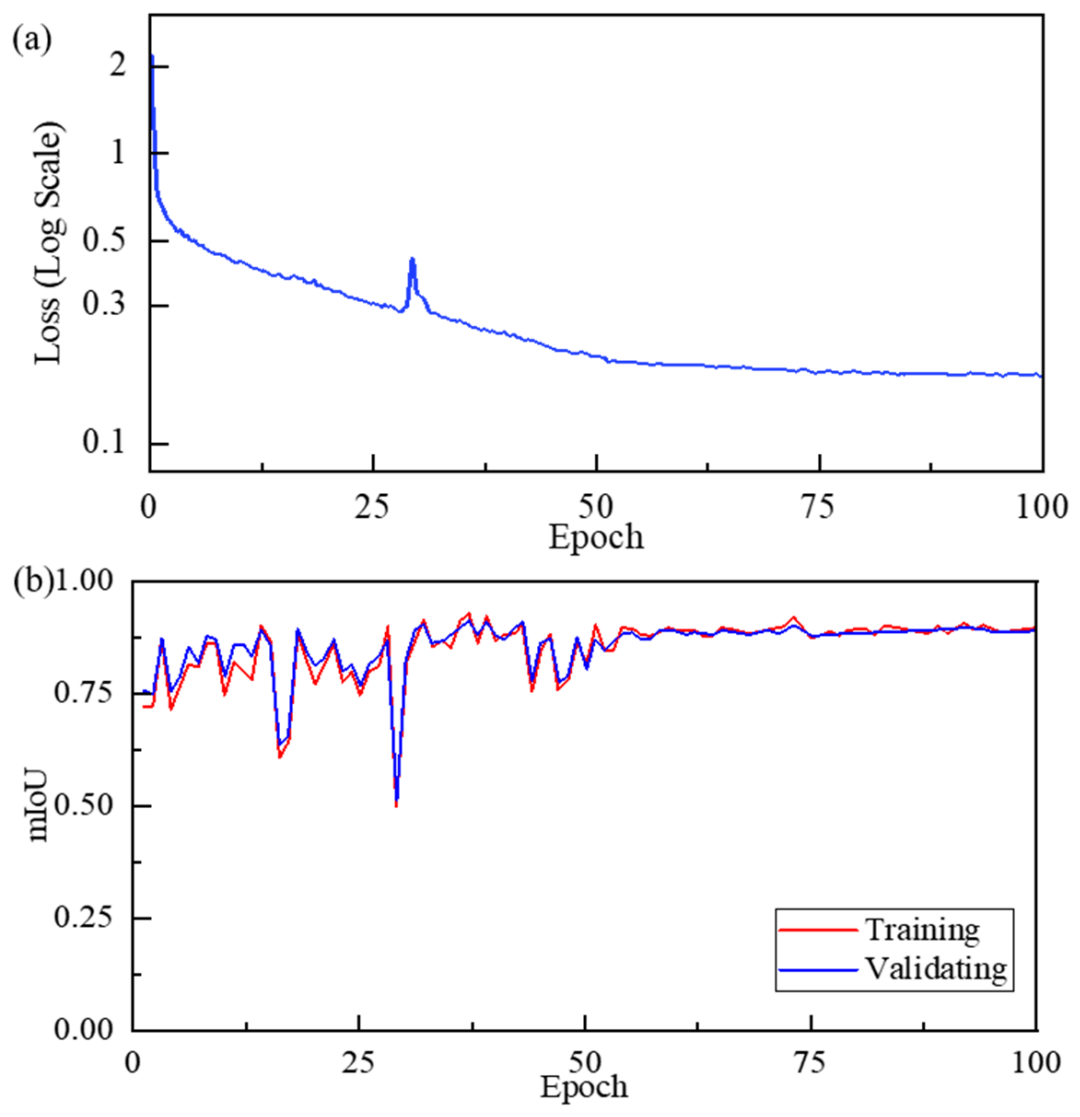
Training curves obtained from the 100-epoch run with a fixed random seed and reconstructed from the DLT interface: (**a**) loss curve and (**b**) mIoU curves for the training and validation sets.

To characterize model convergence under the reported training configuration, the variations in loss and mean intersection over union (mIoU) were recorded over the 100-epoch training run, as shown in Figure 8. Because the commercial training software did not support the direct export of raw training logs or vector-format curves, the curves in Figure 8 were digitized and redrawn from the native curves displayed in the software interface.

As shown in Figure 8a, the loss value decreased rapidly in the early training stage, indicating that the model was able to learn the basic features of multiclass stain regions within a relatively short iteration period. As training progressed, the loss continued to decrease and gradually approached a plateau. Although a local fluctuation occurred near epoch 30, the loss resumed a downward trend after the first learning-rate reduction at epoch 50 and subsequently remained at a relatively low level without evident divergence. These results characterize the convergence behavior of this training run under the reported configuration.

Figure 8b presents the mIoU curves for the training and validation sets. Both curves fluctuated during the early stage of training and gradually stabilized after the learning rate was reduced at epochs 50 and 75. During the later stage, no sustained divergence between the training and validation mIoU curves indicative of overfitting was observed. However, this observation was based on a single training run with a fixed random seed and does not demonstrate performance consistency across repeated runs.

## 4. Results and Discussion

The proposed method was evaluated from five complementary perspectives: overall quantitative performance; class-wise error patterns across blood stains, chemically developed urine stains, and medication stains; qualitative segmentation behavior under different textile substrates and stain morphologies; the contribution of DMACA-based color development to dried urine-stain detection; and computational efficiency and application considerations. 

### 4.1. Overall Quantitative Performance and Class-Wise Error Analysis

Table 4 summarizes the segmentation performance on the independent test set. The model achieved an mIoU of 89.88%, a mean F1 score of 94.57%, and an FWIoU of 98.39%. These values were obtained from a single complete training run with the random seed fixed at 1 and therefore do not represent averages across repeated independent runs.

All values were obtained from a single complete training run with the random seed fixed at 1 and were evaluated on the independent test set. Because repeated training with different random seeds was not performed, across-run standard deviations and confidence intervals were not calculated.

The background class achieved an IoU of 99.11%, indicating that most background pixels were correctly classified despite interference from warp and weft textures, stitching seams, and wrinkle-induced shadows. The blood-stain and medication-stain classes achieved IoU values of 88.11% and 89.62% and F1 scores of 93.68% and 94.53%, respectively. In particular, the medication-stain recall reached 97.16%, indicating that most medication-stain pixels in the test set were detected. Although the local contrast of blood stains was reduced on dark green surgical gowns, the corresponding F1 score remained 93.68%, suggesting that the model retained useful morphological and boundary information under the evaluated low-contrast conditions.

The chemically developed urine-stain class achieved an IoU of 82.67% and an F1 score of 90.51%, which were lower than those of the blood-stain and medication-stain classes. This difference may be associated with the penetration and diffusion of urine within textile fibers. As urine spreads from the stain center toward the periphery, it forms a gradually fading transition region. Some peripheral boundaries therefore remain visually indistinct after color development and may merge with the textile background.

This effect was more apparent in dark green surgical gown samples, where the contrast between the orange–red developed region and the textile substrate was reduced, particularly along weakly developed peripheral boundaries. Consequently, edge pixels were more likely to be classified as background. The lower performance of the chemically developed urine-stain class may therefore result from the combined effects of diffuse boundaries and reduced local contrast on dark substrates.

**Figure 9:**
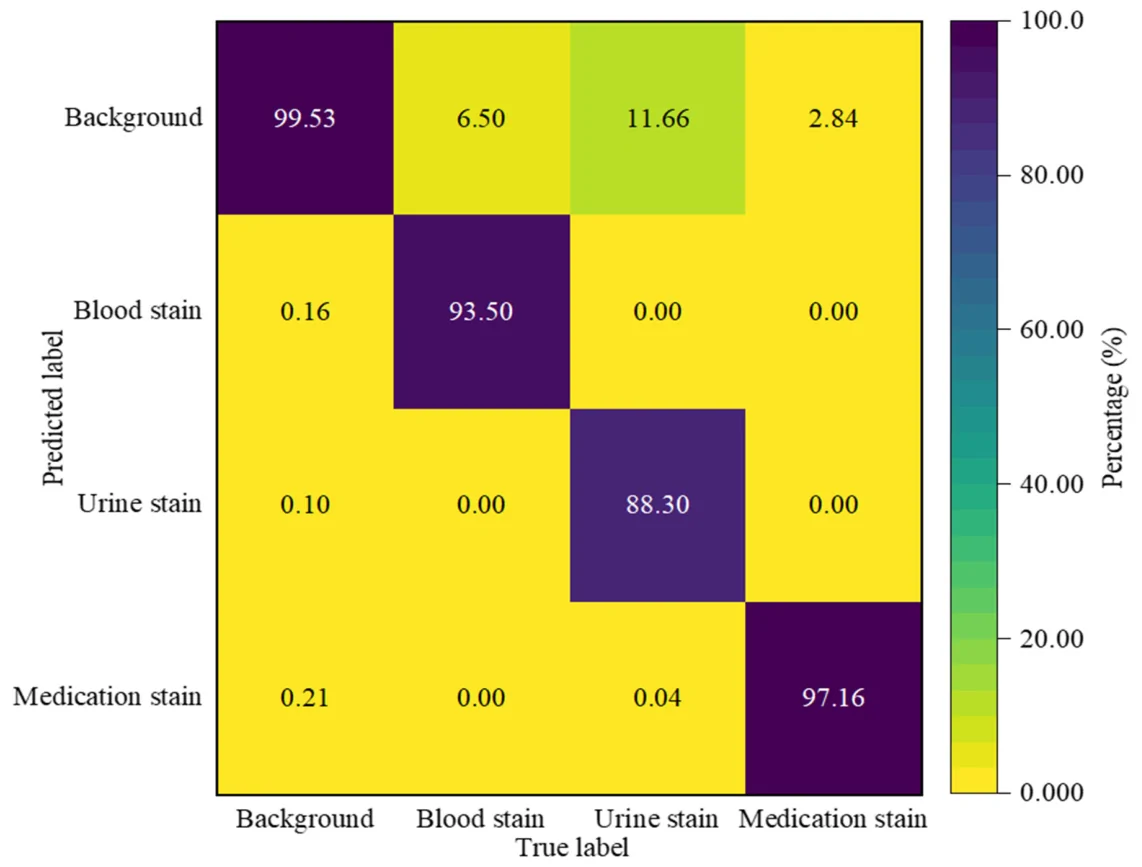
Column-normalized confusion matrix for pixel-level multiclass medical textile stain segmentation on the independent test set. Rows represent predicted classes, columns represent ground-truth classes, and each column is normalized to 100%.

To further identify the sources of segmentation errors, a column-normalized pixel-level confusion matrix was constructed from the independent test-set predictions, as shown in Figure 9.

Direct confusion among the three stain categories was limited. Only 0.04% of the ground-truth chemically developed urine-stain pixels were classified as medication stains, while almost no direct confusion occurred between the other foreground classes. Thus, the model distinguished the three stain categories in most evaluated regions.

The main errors occurred at the interfaces between the stain regions and the background. Specifically, 6.50%, 11.66%, and 2.84% of the ground-truth blood-stain, chemically developed urine-stain, and medication-stain pixels, respectively, were classified as background. These results indicate that missed detection in weak-gradient boundary regions, rather than confusion among stain categories, was the primary source of segmentation error. This finding is consistent with the penetration, diffusion, and drying behavior of liquid contaminants on textile surfaces, which produces gradually fading peripheral regions without sharp color or grayscale transitions.

Among the foreground classes, chemically developed urine stains exhibited the highest background-misclassification rate. Their diffuse peripheral regions and relatively weak optical response after color development made the outer boundaries more likely to merge with the textile substrate. Future work will therefore investigate boundary-aware or edge-focused loss functions to increase the contribution of weak-gradient peripheral pixels during training. Lightweight morphological post-processing will also be evaluated to connect locally discontinuous predictions and recover small missing regions while preserving irregular stain morphology.

For the background class, 99.53% of the ground-truth pixels were correctly classified, and the proportion misclassified as any individual foreground class did not exceed 0.21%. This result indicates that the model produced relatively few false-positive responses to the evaluated textile textures, wrinkle-induced shadows, and stitching seams.

Reduced target–background contrast on dark textiles may also be addressed through wavelength-selective imaging. The spectral responses of DMACA-developed urine-stain regions and different textile substrates could first be measured, after which candidate wavelength bands with greater target–background separability could be selected. Previous research has shown that characteristic absorption or reflection bands and narrow-band image fusion can improve target–background contrast [28]. However, because the target materials and imaging bands investigated in that study differ from those examined here, the effective wavelength bands for chemically developed urine stains on medical textiles require dedicated spectral measurements and comparative experiments.

### 4.2. Qualitative Visualization of Segmentation Results

Representative samples from the independent test set were selected to qualitatively examine segmentation performance across different textile substrates and stain morphologies. For each sample, the input image, manually annotated ground-truth mask, and predicted mask were displayed side by side.

**Figure 10:**
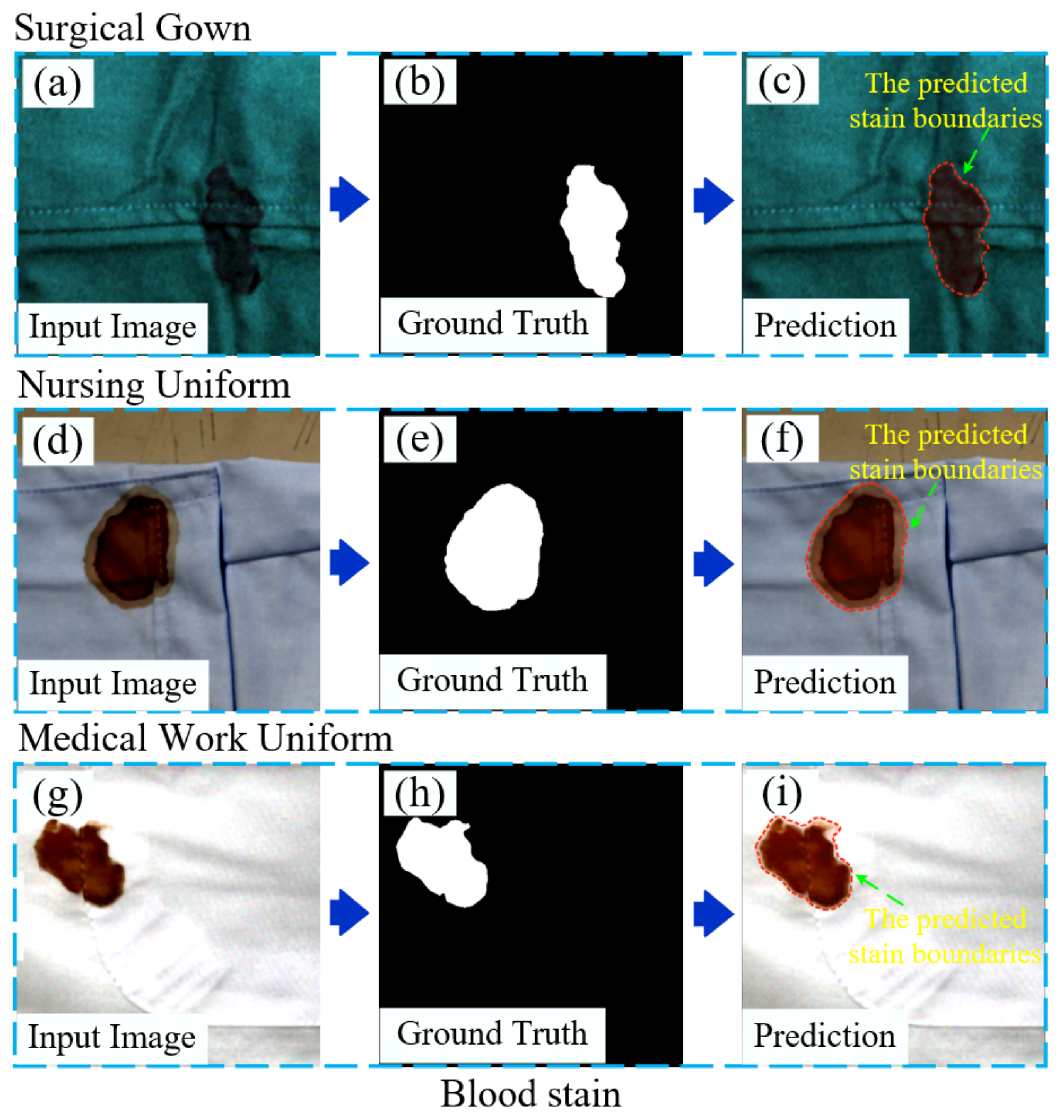
Representative blood-stain segmentation results on different textile substrates. Panels (**a**–**c**), (**d**–**f**), and (**g**–**i**) correspond to the green surgical gown, blue nursing uniform, and white medical work uniform, respectively. Within each group, the panels from left to right show the input image, ground-truth mask, and predicted mask, respectively.

For blood stains, the dark green surgical gown sample in Figure 10a–c exhibited relatively low target–background contrast. Nevertheless, the predicted mask covered most of the main stain region and was generally consistent with the ground-truth annotation. In the light blue nursing uniform sample shown in Figure 10d–f, blood diffusion along the textile fibers produced a visible peripheral serum halo. The model detected both the central stain region and most of the diffused boundary. In the white medical work uniform sample shown in Figure 10g–i, the blood stain exhibited higher contrast, and the predicted main region closely matched the ground-truth mask. A slight outward expansion remained at the boundary, possibly because weak peripheral diffusion pixels were detected by the model but were not fully included in the manual annotation.

**Figure 11:**
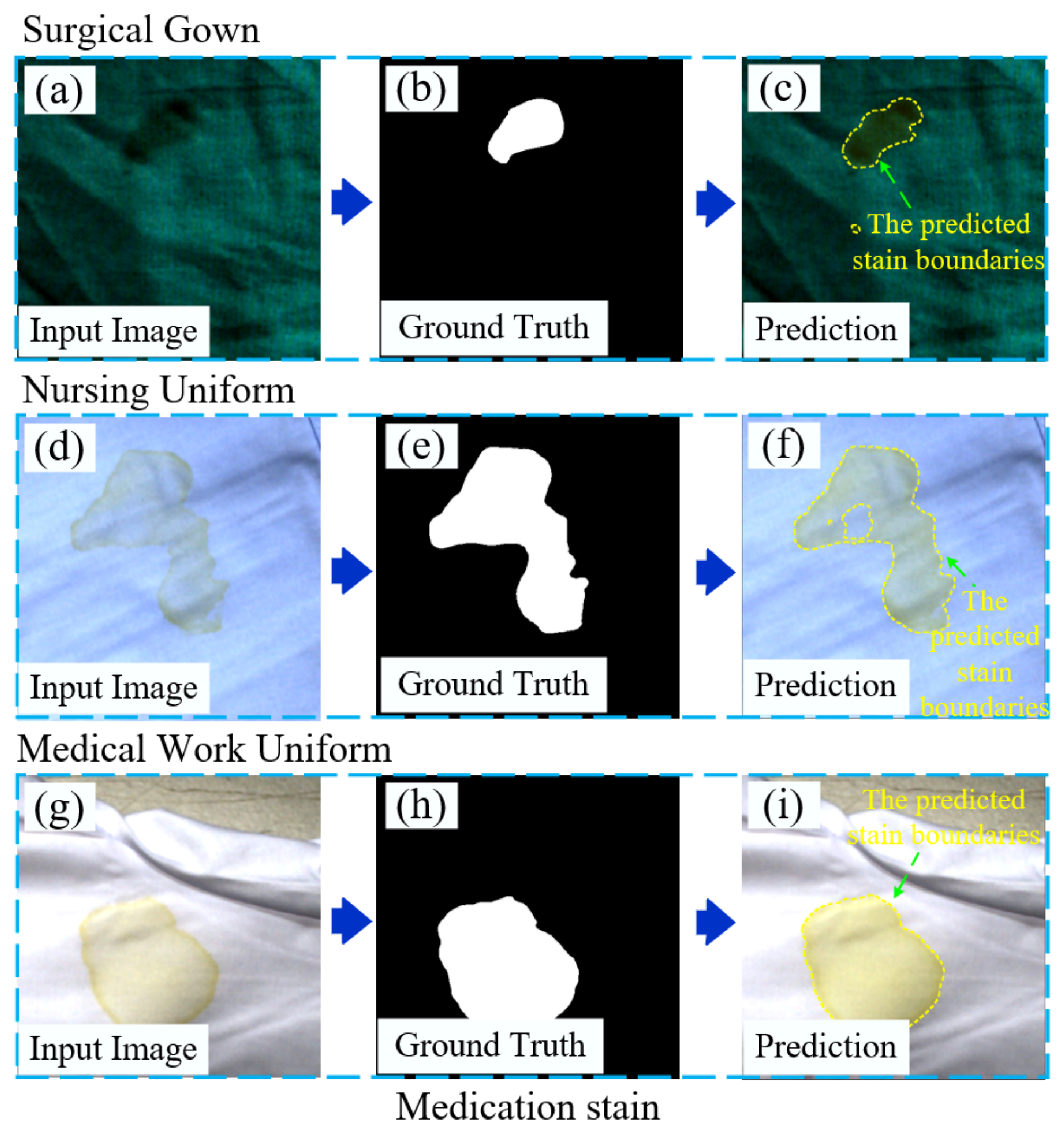
Representative medication-stain segmentation results on different textile substrates. Panels (**a**–**c**), (**d**–**f**), and (**g**–**i**) correspond to the green surgical gown, blue nursing uniform, and white medical work uniform, respectively. Within each group, the panels from left to right show the input image, ground-truth mask, and predicted mask, respectively.

For medication stains, the dark green surgical gown sample in Figure 11a–c appeared as a low-contrast dark region. The model localized most of the stain, although small missed areas and isolated false-positive regions remained. These errors may be associated with the nonuniform stain concentration, weak local gradients, and visually similar background features. In the light blue nursing uniform sample shown in Figure 11d–f, the medication stain displayed darker edges and a lighter center, resembling a coffee-ring morphology. The predicted outer boundary was generally consistent with the manual annotation. In the white medical work uniform sample shown in Figure 11g–i, the stain exhibited relatively high contrast and most of the contaminated region was correctly segmented, although slight deviations remained in weakly stained transition areas.

**Figure 12:**
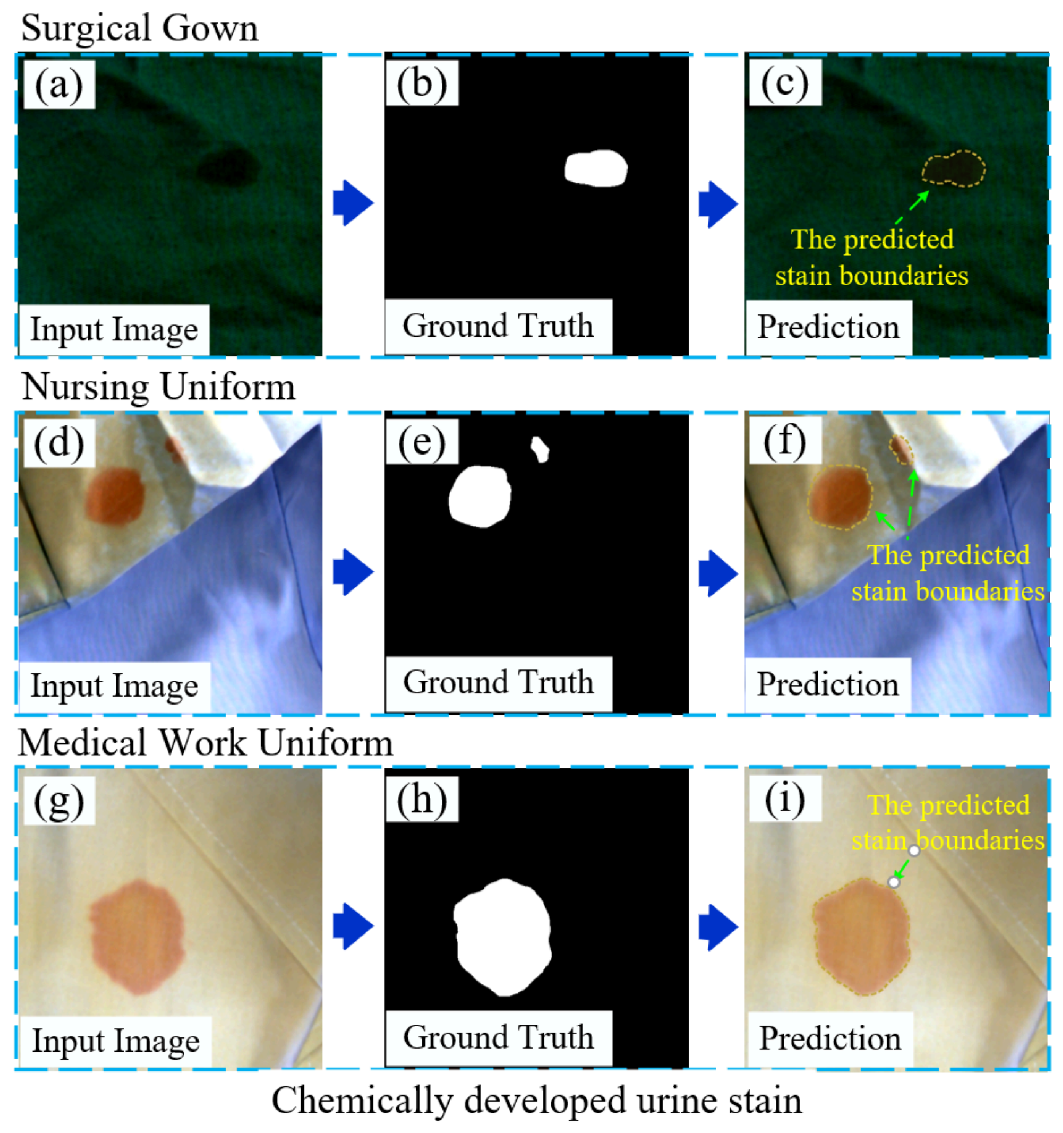
Representative chemically developed urine-stain segmentation results on different textile substrates. Panels (**a**–**c**), (**d**–**f**), and (**g**–**i**) correspond to the green surgical gown, blue nursing uniform, and white medical work uniform, respectively. Within each group, the panels from left to right show the input image, ground-truth mask, and predicted mask, respectively.

For chemically developed urine stains, the dark green surgical gown sample in Figure 12a–c exhibited relatively low contrast between the orange–red developed region and the textile substrate. Nevertheless, most of the main urine-stain region was segmented, indicating that DMACA-based color development provided detectable visual information. In the light blue nursing uniform sample shown in Figure 12d–f, the developed region exhibited clear contrast against the background. The model detected both the main stain region and several spatially separated spots produced by liquid splashing. In the white medical work uniform sample shown in Figure 12g–i, the chemically developed urine stain was clearly distinguishable from the background. The predicted mask was generally consistent with the ground-truth annotation and captured most of the irregular peripheral diffusion region.

Overall, the qualitative results were consistent with the quantitative findings. Stains on light-colored substrates generally exhibited clearer boundaries and more complete segmentation, whereas the principal errors on dark substrates were concentrated in weak-gradient peripheral regions. The results also show that DMACA-based color development supplied detectable visual features for dried urine stains that otherwise lacked sufficient contrast under conventional visible-light imaging.

### 4.3. Ablation Study on Chemical Color Development for Latent Urine Stain Detection

**Figure 13:**
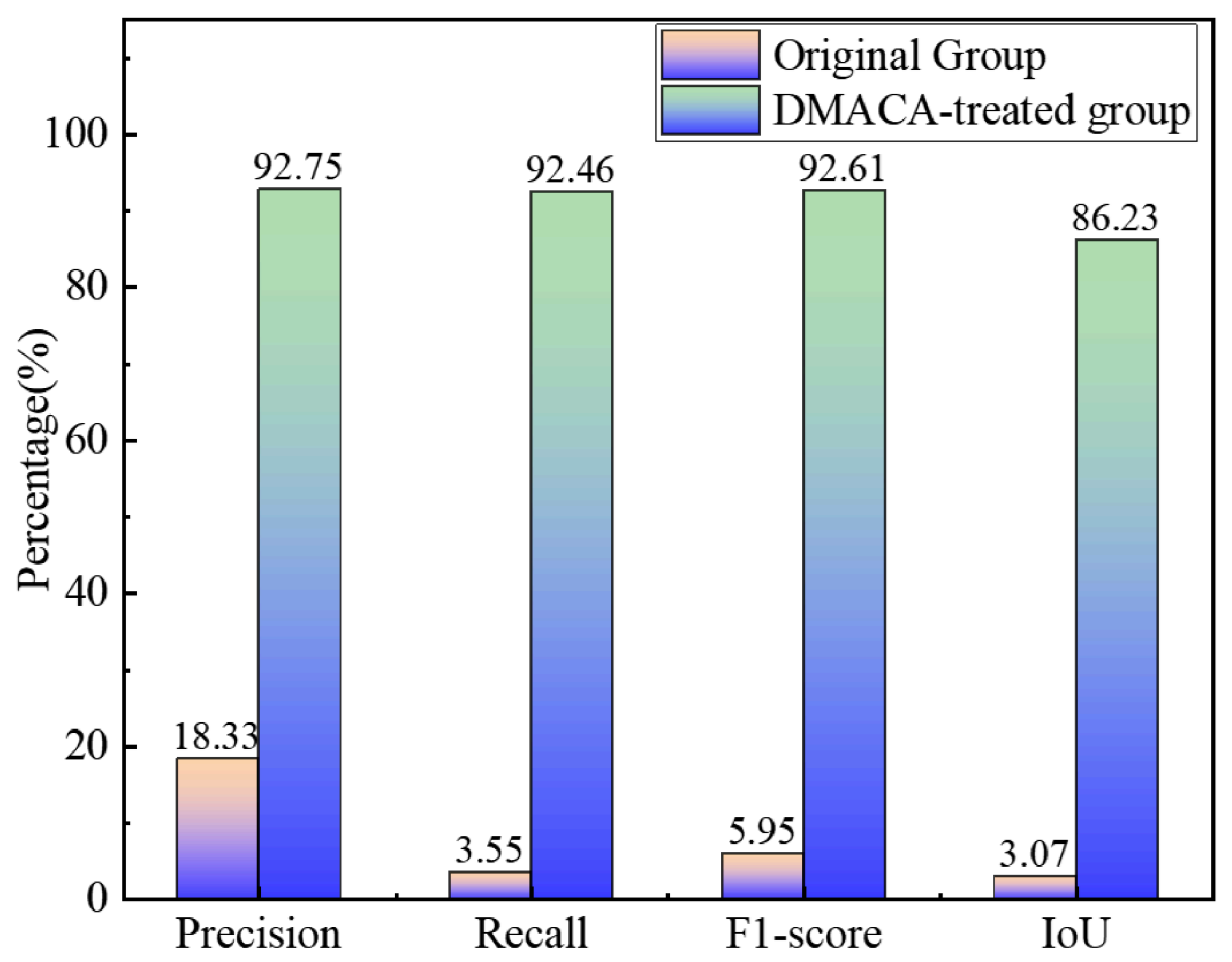
Ablation comparison of precision, recall, F1 score, and IoU for latent urine stains before and after DMACA-based chemical color development.

To assess the contribution of DMACA-based color development at the input stage, urine-stain images acquired before and after chemical treatment were evaluated using the same trained Enhanced semantic segmentation model. The remaining evaluation conditions and preprocessing procedures were kept consistent. The quantitative comparison is shown in Figure 13.

Before chemical color development, segmentation performance was limited, with a precision of 18.33%, a recall of 3.55%, an F1 score of 5.95%, and an IoU of 3.07%. These results indicate that dried urine stains substantially overlapped with the textile background under conventional visible-light imaging and lacked sufficiently discriminative color, texture, and boundary information. Under such conditions, back-end feature learning alone could not compensate for the insufficient optical information in the input images.

After DMACA-based color development, the precision, recall, F1 score, and IoU increased to 92.75%, 92.46%, 92.61%, and 86.23%, respectively. Chemical treatment therefore increased the visual difference between the urine-stained regions and the textile background and transformed weakly visible targets into features that could be identified by the segmentation model. This comparison supports the effectiveness of combining front-end chemical feature enhancement with back-end deep semantic segmentation. Under the visible-light imaging conditions and model configuration evaluated in this study, chemical color development was a critical prerequisite for the effective segmentation of dried urine stains.

### 4.4. Computational Efficiency and Application Considerations

Under the reported hardware configuration, image preprocessing and network inference required an average of 15.39 ms per image on the independent test set. This value represents only the computational stages evaluated in this study and should not be interpreted as the total cycle time of an automated pre-wash sorting system. A complete workflow would additionally include image acquisition, reagent application, color-development waiting, textile positioning and transport, and mechanical sorting.

DMACA-based color development improved the visibility of dried urine stains, while the semantic segmentation model generated pixel-level masks of the predicted contaminated regions. These masks provide spatial information that may support future textile sorting, contamination assessment, and washing-process selection. However, their effectiveness in a complete hospital laundry workflow has not yet been experimentally validated.

The present study primarily evaluated individually prepared stain categories. In practical hospital laundry operations, urine, blood, iodophor, detergent residues, and other contaminants may coexist or overlap. Although the DMACA response is expected to be primarily associated with urine-related components, direct cross-reactivity with other contaminants was not experimentally evaluated. Coexisting substances may also alter the intensity and spatial distribution of the developed color through color masking, local acidity changes, and modified wetting or diffusion behavior. Therefore, cross-reactivity, class confusion, and segmentation performance under mixed-contaminant conditions require systematic investigation.

The experiments were also mainly conducted using single-layer, relatively flat textile samples that had been naturally dried for 24 h. The effects of severe wrinkles, local moisture, textile overlap, and different post-development intervals, such as 30 min, were not systematically evaluated. Future studies will investigate these complex physical conditions, the temporal stability of the DMACA-induced color response, mixed contaminants, and complete workflow integration to further assess segmentation consistency and practical applicability.

## 5. Conclusions

This study developed a multiclass medical textile stain detection method integrating front-end chemical color development with back-end deep semantic segmentation to address the blurred boundaries of visually apparent stains and the insufficient visual features of dried urine stains. DMACA-based color development transformed urine stains that were difficult to distinguish under conventional visible-light imaging into orange–red visual features. Based on the spatial color difference ΔE in the L*a*b* color space, 0.0183 mol/L was selected as the most suitable DMACA concentration among those tested, providing a favorable balance between color contrast and response stability.

A dataset of 1974 images was constructed using white medical work uniforms, blue nursing uniforms, and green surgical gowns. It included blood stains, chemically developed urine stains, medication stains and uncontaminated textiles. The IoU values for blood stains, chemically developed urine stains, and medication stains were 88.11%, 82.67%, and 89.62%, respectively. The combined image-preprocessing and network-inference time averaged 15.39 ms per image. In the ablation comparison, DMACA-based color development increased the urine-stain IoU from 3.07% to 86.23%, confirming its substantial contribution to the effective segmentation of dried urine stains. These results support the feasibility of combining front-end chemical feature enhancement with deep semantic segmentation for pixel-level stain recognition under the current dataset and experimental conditions.

Several limitations remain. The reported metrics were obtained from a single training run with a fixed random seed. Repeated training with multiple random seeds, benchmark comparisons with other segmentation models, and independent external validation were not conducted. Weak-gradient boundaries, reduced local contrast on dark textiles, mixed contaminants, severe wrinkles, local moisture, textile overlap, and the temporal stability of the color response also require further investigation. Future work will include repeated multi-seed experiments, unified multi-model comparisons, boundary-aware segmentation, wavelength-selective imaging, diverse external datasets, and workflow-level validation to further evaluate the stability, generalization, and practical applicability of the proposed method.

## Figures and Tables

**Table 1:** Spatial color difference (ΔE) of urine-stained regions under different DMACA concentrations.

Group	Sample 1	Sample 2	Sample 3	Sample 4	Sample 5	Mean	Standard Deviation
A	5.533	8.102	13.385	11.699	8.021	9.348	3.150
B	14.983	28.775	23.111	16.098	14.549	19.503	6.236
C	25.408	22.264	20.264	25.710	21.025	22.934	2.502
D	37.196	35.325	31.674	35.189	35.789	35.035	2.040
E	36.663	33.234	27.820	40.006	47.276	37.000	7.301
F	23.474	29.046	22.069	27.076	23.083	24.950	2.969

**Table 2:** Composition of the multiclass medical textile stain dataset.

Image Category	Surgical Gown (Green)	Nursing Uniform (Blue)	Work Uniform (White)	Total
Blood stain	188	185	196	569
Medication stain	182	212	198	592
Chemically developed urine stain	174	220	205	599
Uncontaminated textile	61	75	78	214
Total	605	692	677	1974

**Table 3:** Hyperparameter settings for deep semantic segmentation network training.

Parameter	Setting
Dataset split	Training/validation/test = 70%/15%/15%
Pretrained model	HALCON Enhanced semantic segmentation pretrained model
Number of epochs	100
Number of iterations	8700
Batch size	16
Solver type	Adam
Initial learning rate	1 × 10^−4^
Learning rate schedule	Reduced to 1 × 10^−5^ at epoch 50 and to 1 × 10^−6^ at epoch 75
Weight decay	5 × 10^−4^
Random seed	1
Number of independent training runs	1
Input image size	512 × 512 × 3
Final evaluation subset	Independent test set

**Table 4:** Class-wise segmentation performance for medical textile stains.

Segmentation Class	Precision (%)	Recall (%)	F1 Score (%)	IoU (%)
Background	99.58	99.53	99.55	99.11
Blood stain	93.86	93.50	93.68	88.11
Chemically developed urine stain	92.83	88.30	90.51	82.67
Medication stain	92.03	97.16	94.53	89.62

## Data Availability

The original contributions presented in this study are included in the article. Further inquiries can be directed to the corresponding authors.

## Appendix A. Details of DMACA Concentration Preparation and Spatial Color Difference Calculation

To obtain a stable color development effect suitable for machine vision detection, the concentration of the DMACA reagent was further optimized quantitatively. Considering the safety requirements of pre-wash medical textile inspection and the tolerance of the fabric substrate, a mild mixed-solvent system was used to prepare the reagent. The total volume of the system was 5.0 mL, consisting of 3.5 mL of 95% ethanol, 0.5 mL of distilled water, and 1.0 mL of 99.8% glacial acetic acid. During reagent preparation, a microanalytical balance with an accuracy of 0.001 g was used, and the actual amount of DMACA added was measured using the dropper differential weighing method. The mass was then converted into molar concentration. Six concentration gradients were designed, and each gradient contained five independent replicate samples (*n* = 5). The color development process was designed to simulate practical spraying conditions, followed by natural air drying and standing for 10 min at room temperature. The molar concentration was calculated according to Equation (A1), and the detailed preparation results are listed in Table A1.

(A1) $$c=\frac{n}{V}=\frac{m}{M\times V}$$

where n is the amount of substance (mol), m is the actual net mass of DMACA measured by the dropper differential weighing method (g), M is the molar mass of DMACA (175.23 g/mol), c is the molar concentration (mol/L), and V is the total constant volume of the mixed-solvent system (L).

**Table A1:** Quantitative preparation of DMACA reagent concentration gradients.

Group	Initial Dropper Weight (g)	Final Dropper Weight (g)	Net Mass (g)	Molar Concentration (mol/L)
A	0.765	0.762	0.003	0.0034
B	0.767	0.760	0.007	0.0080
C	0.771	0.763	0.008	0.0091
D	0.776	0.760	0.016	0.0183
E	0.780	0.761	0.019	0.0217
F	0.784	0.760	0.024	0.0274

To reduce the influence of ambient illumination fluctuations and camera sensor baseline drift on measurement results, an in-image blank calibration method was used to evaluate the color development response. A urine color-developed region and a pure-reagent blank region were prepared simultaneously on the same fabric substrate and within the same imaging field of view, and images were captured synchronously using the industrial camera. Considering that edge diffusion during color development may lead to unstable contours, contour segmentation based on stain edges was not used during feature extraction. Instead, fixed circular regions of interest (ROIs) were set at the center of the color-developed region and in the pure-reagent background region. The target ROI was selected from the orange–red urine-stained region after color development, while the background ROI was selected from the pure-reagent region in the same image for comparison.

The mean values of the L*, a*, and b* channels were then extracted from the two types of ROIs, and the spatial color difference ΔE was calculated to quantify the color separation between the color-developed region and the background region. A larger ΔE indicates a more pronounced color difference between the target and background, whereas a smaller standard deviation indicates a more stable color development response. The spatial color difference, mean value, and standard deviation were calculated according to Equations (A2)–(A4).

(A2) $$\Delta E=\sqrt{{\left(L_{target}^{*}-L_{bg}^{*}\right)}^{2}+{\left(a_{target}^{*}-a_{bg}^{*}\right)}^{2}+{\left(b_{target}^{*}-b_{bg}^{*}\right)}^{2}}$$

(A3) $$\bar{x}=\frac{1}{N}\sum_{i=1}^{N}x_{i}$$

(A4) $$S=\sqrt{\frac{1}{N-1}\sum_{i=1}^{N}{\left(x_{i}-\bar{x}\right)}^{2}}$$

where $L_{target}^{*}$, $a_{target}^{*}$, $b_{target}^{*}$ and $L_{bg}^{*}$, $a_{bg}^{*}$, $b_{bg}^{*}$ denote the mean values of the lightness, red–green, and yellow–blue channels extracted from the orange–red urine color-developed region and the pure-reagent background region in the L*, a*, and b* color space. The subscripts target and bg represent the orange–red urine color-developed region and the pure-reagent background region in the same image, respectively. $\bar{x}$ is the mean spatial color difference of all samples, N is the number of replicate samples under a single concentration gradient, $x_{i}$ is the spatial color difference of the i-th independent sample, and S is the sample standard deviation.
